# Differences in the gut microbiota of dogs (*Canis lupus familiaris*) fed a natural diet or a commercial feed revealed by the Illumina MiSeq platform

**DOI:** 10.1186/s13099-017-0218-5

**Published:** 2017-11-21

**Authors:** Junhyung Kim, Jae-Uk An, Woohyun Kim, Soomin Lee, Seongbeom Cho

**Affiliations:** BK21 PLUS Program for Creative Veterinary Science Research, Research Institute for Veterinary Science and College of Veterinary Medicine, Seoul National University, Bldg 85, Suite #731, 1 Gwanak-ro, Gwanak-gu, Seoul, 08826 Republic of Korea

**Keywords:** *Canis lupus familiaris*, Natural diet, Next-generation sequencing, Gut microbiota

## Abstract

**Background:**

Recent advances in next-generation sequencing technologies have enabled comprehensive analysis of the gut microbiota, which is closely linked to the health of the host. Consequently, several studies have explored the factors affecting gut microbiota composition. In recent years, increasing number of dog owners are feeding their pets a natural diet i.e., one consisting of bones, raw meat (such as chicken and beef), and vegetables, instead of commercial feed. However, the effect of these diets on the microbiota of dogs (*Canis lupus familiaris*) is unclear.

**Methods and results:**

Six dogs fed a natural diet and five dogs fed a commercial feed were selected; dog fecal metagenomic DNA samples were analyzed using the Illumina MiSeq platform. Pronounced differences in alpha and beta diversities, and taxonomic composition of the core gut microbiota were observed between the two groups. According to alpha diversity, the number of operational taxonomic units, the richness estimates, and diversity indices of microbiota were significantly higher (*p* < 0.05) in the natural diet group than in the commercial feed group. Based on beta diversity, most samples clustered together according to the diet type (*p* = 0.004). Additionally, the core microbiota between the two groups was different at the phylum, family, and species levels. Marked differences in the taxonomic composition of the core microbiota of the two groups were observed at the species level; *Clostridium perfringens* (*p* = 0.017) and *Fusobacterium varium* (*p* = 0.030) were more abundant in the natural diet group.

**Conclusions:**

The gut microbiota of dogs is significantly influenced by diet type (i.e., natural diet and commercial feed). Specifically, dogs fed a natural diet have more diverse and abundant microbial composition in the gut microbiota than dogs fed a commercial feed. In addition, this study suggests that in dogs fed a natural diet, the potential risk of opportunistic infection could be higher, than in dogs fed a commercial feed. The type of diet might therefore play a key role in animal health by affecting the gut microbiota. This study could be the basis for future gut microbiota research in dogs.

**Electronic supplementary material:**

The online version of this article (10.1186/s13099-017-0218-5) contains supplementary material, which is available to authorized users.

## Background

The gut microbiota is the collection of living microorganisms inhabiting the gastrointestinal (GI) tract of the host. It has been estimated that the gut microbiota of humans and animals consists of 10^10^–10^14^ microbial cells, a number roughly similar or 10 times higher than the number of host cells [1, 2].

In the past, studies on the microbiota were performed using culture-based methods, and research in the field was limited. However, recent advances in next-generation sequencing (NGS) technologies have allowed a more comprehensive analysis of the complex and diverse gut microbial communities [3]. Therefore, the number of studies on the gut microbiota using NGS system, which could provide a broad and deep understanding of the microbiota, is on the rise. These studies have revealed that the gut microbiota is closely linked with the host’s health and disease status, including maintenance of the GI health, stimulation of the immune system, development of obesity, and various GI disorders, including inflammatory bowel disease [4, 5]. Concomitant with the analysis of the relationship between the microbiota and health, several studies are aiming to identify the various factors affecting the microbiota [6]. Some studies have suggested that among such diverse factors, the diet greatly influences the composition of the gut microbiota; hence, recent studies have largely focused on the relationship between the diet and gut microbiota. The results revealed that animal-based diets might cause an increase in the abundance of *Alistipes*, *Bilophila*, and *Bacteroides* at the genus level, and a decrease in the abundance of *Firmicu*tes at the phylum level in the human gut microbiota; furthermore, the human gut microbiota has been divided into a *Prevotella* enterotype and a *Bacteroides* enterotype, according to long-term diet [7, 8].

The dog (*Canis lupus familiaris*) is one of the closest companion animals to humans; over the years, as the quality of life improved, the number of people who raised dogs increased. In addition, people began treating their dogs as family members rather than pets, and began focusing on their health [9]. However, gut microbiota studies were mainly focused on human and human-oriented mouse models [4–8]. Several studies of the dog were conducted, including comparisons of the microbiota of obese and lean dogs; comparisons of the microbiota in the presence or absence of GI diseases; and comparisons of the microbiota according to the presence or absence of dietary fiber or boiled meat in the diet [10–13]. Nevertheless, most of these studies of dog gut microbiota were performed using the 454 pyrosequencing techniques, which are rarely used now-a-days; furthermore, they were not nearly as numerous as human-based studies, and did not match the increase in the numbers of pet owners, and their increased interest in dog health.

In recent years, the number of pet owners who feed their dogs a natural diet, i.e., one consisting of bones, raw meat (such as chicken and beef), and vegetables, instead of commercial feed, has increased [14]. This increase was fueled by the 2007 pet food recalls, because of melamine contamination [15]. In parallel with this trend, the advantages and disadvantages of a natural diet were discussed. Feeding dogs a natural diet was associated with some health benefits, such as fresher breath, healthier coat and skin, alleviation of arthritis, and improved immune response [16]. In contrast, some studies have provided evidences that discourage the use of natural diet because of nutritional imbalance and bacterial contamination. Feeding dogs a natural diet led to a pronounced nutritional imbalance and increased the risk of exposure to zoonotic pathogenic bacteria, including *Salmonella* spp., *Campylobacter* spp., and pathogenic *Escherichia coli*, which threaten the dog and public health [14, 15, 17, 18]. These studies, however, were only based on nutrition, pathogen detection, and clinical experience, and there were few studies about changes in microbiota composition associated with natural diet and commercial feed in dogs [19].

Given the above, factors that can influence the gut microbiota, especially diet type, in dogs, need to be studied. The current study was performed to investigate the effect of long-term diet, i.e., natural diet and commercial feed, on the gut microbiota. Specifically, (1) we identified the core microbiota of dogs fed a natural diet or a commercial feed up to the species level; and (2) compared the differences in alpha diversity, beta diversity, and the composition of gut microbiota between animals fed the two different types of diet.

## Methods

### Animals and diets

For this study, 24 dogs were initially recruited; 11 dogs were from a pet owner group (Seoul, Korea), which is a social community of pet owners gathered to feed dogs a natural diet. Pet owners in this group share information of dogs and raise dogs in the same way, walking a dog in a similar way and feeding a natural diet in similar rate of raw meat and vegetables (90% of raw meat: 10% of vegetables). Thirteen dogs belonged to veterinary college (Seoul, Korea) students who fed the dogs a commercial feed. From among these, 11 representative fecal samples (6 dogs fed a natural diet and 5 dogs fed a commercial feed) were selected for gut microbiota analysis according to the selection criteria, which included diet, medical history, living area (indoor), breed, gender, age, and weight. All dogs analyzed in this study were small breeds, including the Maltese (n = 2), Yorkshire terrier (n = 2), Pomeranian (n = 1), Poodle (n = 2), Bichon Frise (n = 3), and white West Highland terrier (n = 1) breeds. The natural diet was based on approximately 90% raw meat (kangaroo, beef, chicken, or duck) and 10% vegetables; the commercial feed comprised Natural Balance (Natural Balance Korea CO., Ltd., Suwon, Kyonggi-Do, Korea) and LAMER Dr. Heal Skin care (CHD MEDICS CO., Ltd., Goyang, Kyonggi-Do, Korea). The chemical composition of these two commercial feed is crude protein (18–21% of total content), crude fat (8–10%), crude fiber (3–5%), and crude ash (7%), and 10% of moisture. The median age of the dogs was 36 months (range 12–144 months); the median body weight was 4.3 kg (range 2.8–8.3 kg); and the body condition score of all dogs was 5, based on a 9-point scale [20]. All dogs were clinically healthy, had not been receiving any medications that could have affected the gut microbiota for at least 6 months prior to the study, and their diet had not been changed for at least 1 year prior to sample collection. Detailed information about the animals and their diet is provided in Table 1.

**Table 1:** Information on the dogs enrolled in this study

Group	Name	Breed	Age (months)	Gender	Weight (kg)	Diet and quantity (g)	Number of meals per day
Natural diet group	ND-1	Poodle	46	SF	4.3	Kangaroo (75 g), vegetables (10 g)	1/day
	ND-2	Poodle	28	CM	5.2	Kangaroo (110 g), vegetables (15 g)	1/day
	ND-3	BF	24	SF	4.5	Beef (90 g), vegetables (10 g)	2/day
	ND-4	WH	12	CM	7	Chicken + duck (150 g), vegetables (10 g)	2/day
	ND-5	BF	36	SF	6.7	Duck (150 g), vegetables (10 g)	1/day
	ND-6	BF	27	CM	8.3	Duck (200 g), vegetables (15 g)	1/day
Commercial feed group	CF-1	Mal	144	F	3	Natural balance	2/day
	CF-2	Mal	94	CM	2.8	LAMER Dr. Heal skin care	1/day
	CF-3	YT	77	CM	4.2	LAMER Dr. Heal skin care	1/day
	CF-4	Pom	36	M	4	Natural balance	2/day
	CF-5	YT	57	SF	3.2	Natural balance	2/day

Natural diet group (ND): dogs fed a natural diet; Commercial feed group (CF): dogs fed a commercial feed

*BF* bichon frise; *WH* white west highland terrier; *Mal* the maltese; *YT* yorkshire terrier; *Pom* pomeranian; *M* male; *CM* castrated male; *F* female; *SF* spayed female

### Sample collection and DNA extraction

Fecal samples of 11 dogs were analyzed. Fresh fecal samples were individually collected, and then immediately transported to the laboratory at 4 °C. All samples were stored at − 75 °C for microbial community analysis. After thawing the frozen fecal samples, metagenomic DNA was extracted using the FastDNA SPIN extraction kit (MP Biomedicals, Santa Ana, CA, USA) following the manufacturer’s instructions. All metagenomic DNA samples were stored at 4 °C for microbial community analysis.

### Polymerase chain reaction (PCR) amplification and Illumina sequencing

For bacterial DNA amplification, the reactions were carried out using the extracted metagenomic DNA, with the primers 341F and 805R, targeting the V3–V4 regions of the *16S rRNA* gene (Additional file 1). A secondary amplification to attach the Illumina Nextera barcodes was then performed using the i5 forward primer and i7 reverse primer (Additional file 1); PCR products were examined via 2% agarose gel electrophoresis. For purification of the amplified products, the QIA quick PCR purification kit (Qiagen, Valencia, CA, USA) was used. Equal amounts of purified products were pooled together, and non-target fragments were removed using the Ampure beads kit (Agencourt Bioscience, Beverly, MA, USA). The product size and quality were evaluated on a Bioanalyzer 2100 (Agilent, Palo Alto, CA, USA). The amplicons were pooled, and sequencing was conducted at Chunlab, Inc. (Seoul, Korea) using an Illumina MiSeq sequencing system (Illumina, San Diego, CA, USA).

### MiSeq pipeline

The raw reads were first submitted to a quality check and filtering of low quality (< Q25) reads by Trimmomatic 0.32. The paired-end sequences (250 bp) were then merged using PandaSeq [21, 22]. Primers were trimmed using Chunlab in-house program (Chunlab, Inc., Seoul, Korea) at a similarity cut-off of 0.8; the sequences were denoised using the DUDE-Seq to correct the sequencing errors. From all quality controlled sequences, 20,000 reads were randomly selected, and UCHIME and the 16S database in the EzBioCloud were used to identify chimera reads with a best hit similarity rate below 97% [23]. Taxonomic assignment was performed based on the EzBioCloud database, and sequence similarity was calculated via pairwise alignment [24, 25]. Sequences that matched the reference sequence by more than 97% similarity in EzBioCloud were considered identified at the species level. The sequences that were not matched to the EzBioCloud 16S database were then clustered using cluster database at high identity with tolerance (CD-HIT) and UCLUST tools with 97% similarity boundary [26, 27]. The species identified at the EzBioCloud 16S database and OTUs obtained by CD-HIT and UCLUST tools were combined to form the final set of OTUs, and the remaining singletons were ignored. Other sequence similarity cut offs were genus (97% > x ≥ 94.5%), family (94.5% > x ≥ 86.5%), order (86.5% > x ≥ 82%), class (82% > x ≥ 78.5%), and phylum (78.5% > x ≥ 75%).

### Data and statistical analyses

The alpha and beta diversities were analyzed by using CL community™ version 3.43 (Chunlab, Inc.). The alpha diversity analysis, including rarefaction curve and diversity indices, was carried out. The beta diversity, including principal coordinate analysis (PCA), was analyzed based on Fast UniFrac [28]. The core microbiota was defined as including microorganisms present in more than 80% of dog feces in each diet group, and at the same time accounting for more than 0.1% of the total microbial community.

Differences in the alpha diversity, including the number of operational taxonomic units (OTUs), richness, and diversity, were investigated in the two diet groups. Furthermore, differences in taxonomic composition between the two groups, from the phylum to species level, were analyzed. Statistical analysis was performed using a Mann–Whitney U test in SPSS statistics package, version 22.0 (SPSS IBM, New York, NY, USA), and a *p* value of < 0.05 was accepted as indicating statistical significance.

## Results

### Sequence analysis

**Fig. 1:**
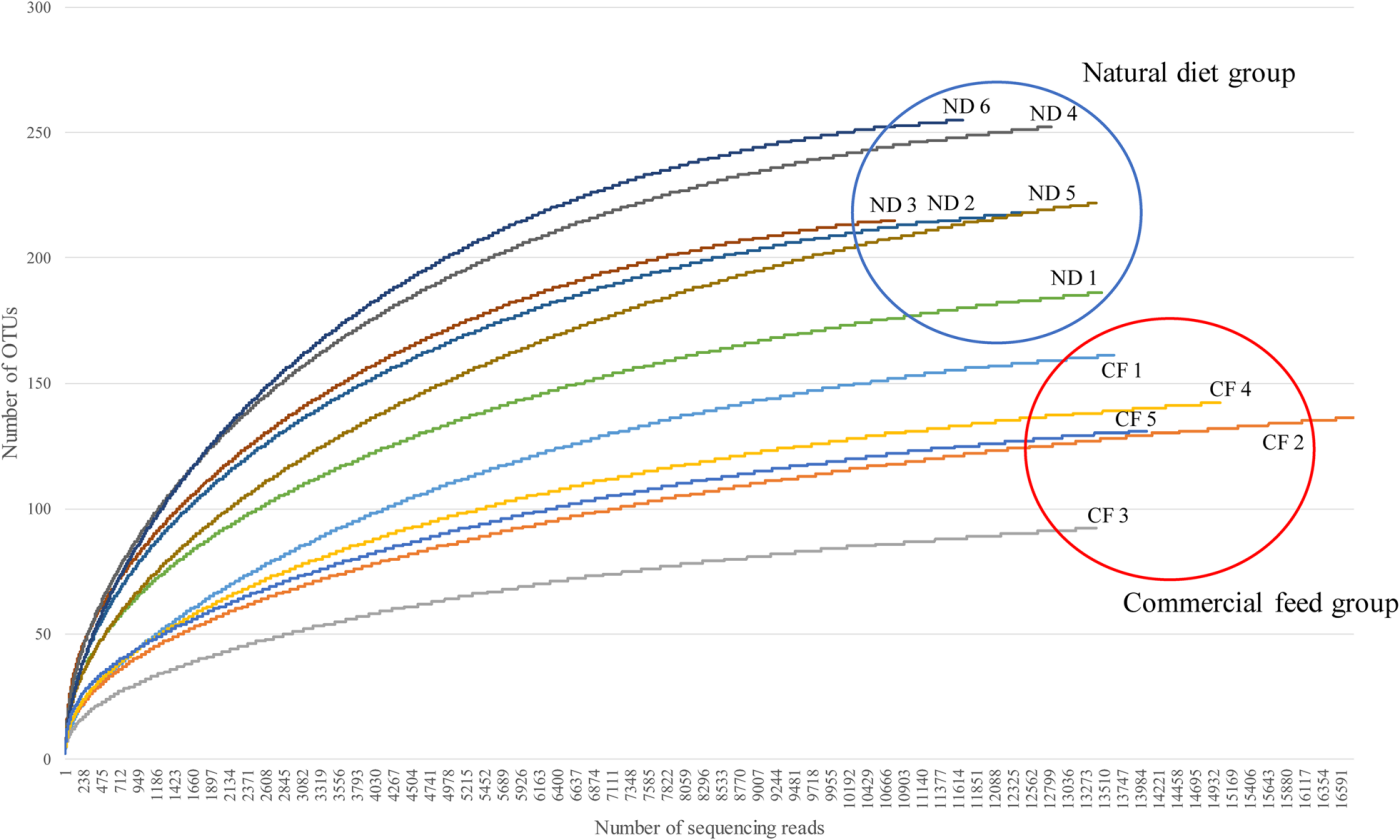
Rarefaction curves for gut microbial communities in 11 dogs. The number of operational taxonomic units (OTUs) in the natural diet (ND) group was higher, while the number of valid reads was lower, than in the commercial feed (CF) group (*p* = 0.004 for both comparisons)

In total, 3288,464 reads were obtained from the fecal samples; 1896,221 were from the natural diet group (median read number 317,576; range 237,356–387,829), and 1392,243 were from the commercial feed group (median read number 284,801; range 224,940–311,212). After quality trimming, merging, primer trimming, and length trimming, 20,000 reads were randomly selected, and chimera reads were removed; 171,495 valid reads were hence obtained from 11 samples. The number of valid reads from the natural diet group was 14593.67 ± 893.73 (mean ± SD), and from the commercial feed group, 16,786.6 ± 1072.53 (*p* = 0.004). The median read length was 409.54 (403.91–419.99) and the median Good’s library coverage was 99.77734 (99.5751–99.81349). An additional file shows these in more detail (Additional file 2). The rarefaction curves for all 11 samples are shown in Fig. 1.

### Alpha diversity

**Fig. 2:**
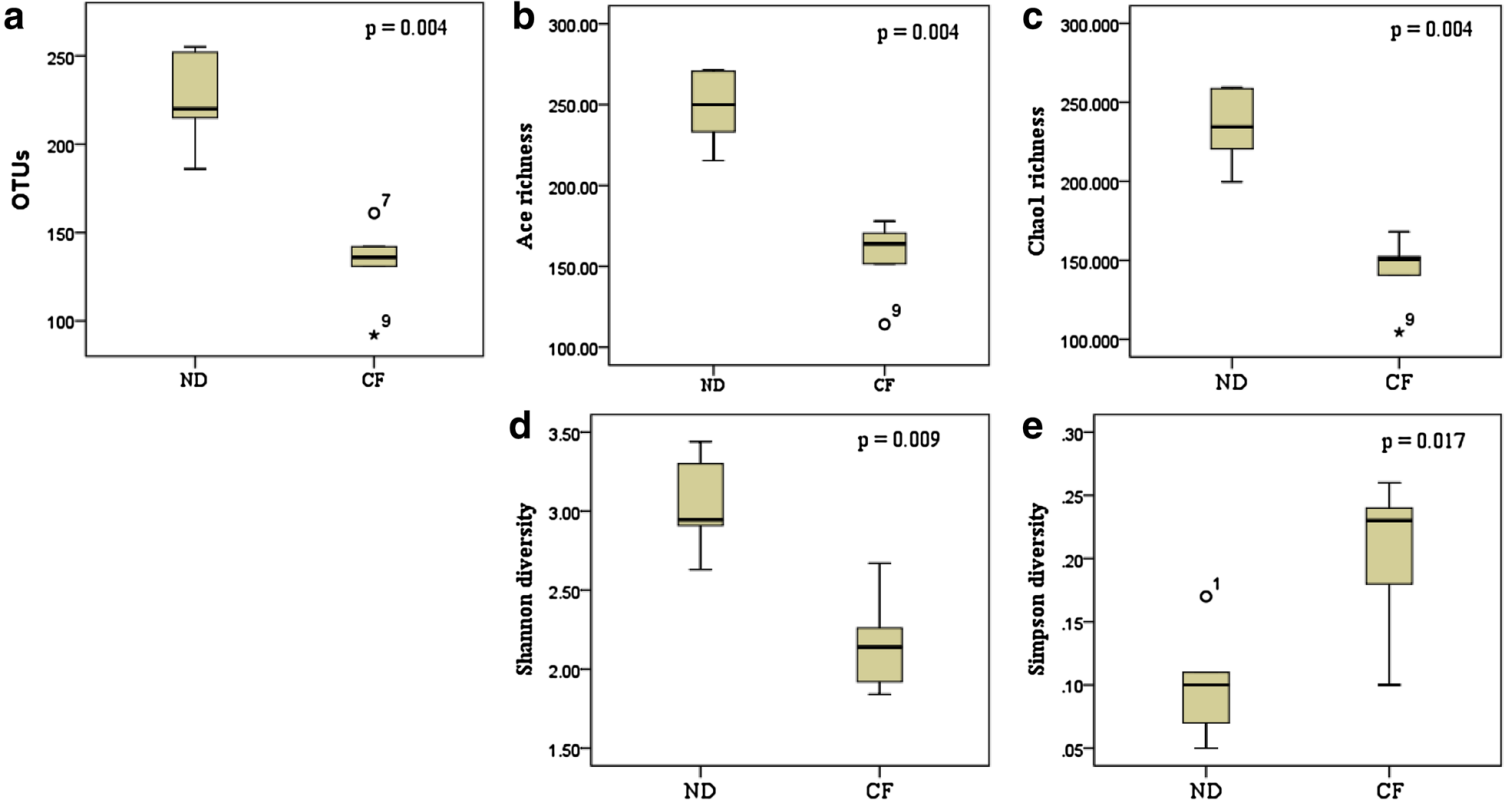
Box plots of the alpha diversity indices in the two diet groups. ND, natural diet group; CF, commercial feed group. Asterisks refer to extreme values, and circles refer to potential outliers. **a** The number of OTUs, **b** Ace richness estimates, **c** Chao1 richness values, **d** Shannon diversity indices, and **e** Simpson diversity indices

The number of OTUs in the natural diet group was significantly higher than that in the commercial feed group (natural diet group: 224.67 ± 25.72, commercial feed group: 132.4 ± 25.28, *p* = 0.004; Fig. 2a). The species richness estimates were significantly higher in the natural diet group than in the commercial feed group. The Ace richness estimates of the natural diet group and commercial feed group were 248.48 ± 23.78 and 155.64 ± 25.13 (*p* = 0.004; Fig. 2b), respectively; the Chao1 richness values were 234.56 ± 23.66 and 143.31 ± 23.81 (*p* = 0.004), respectively (Fig. 2c). In addition, the diversity indices of the natural diet group were also significantly higher than that in the commercial feed group based on the Shannon diversity index and Simpson diversity index. The Shannon diversity indices of the natural diet and commercial feed groups were 3.03 ± 0.29 and 2.17 ± 0.33, respectively (*p* = 0.009; Fig. 2d); the Simpson diversity indices were 0.10 ± 0.04 and 0.20 ± 0.06, respectively (*p* = 0.017; Fig. 2e). The raw data of alpha diversity for all samples are shown in Additional file 2.

### Beta diversity

**Fig. 3:**
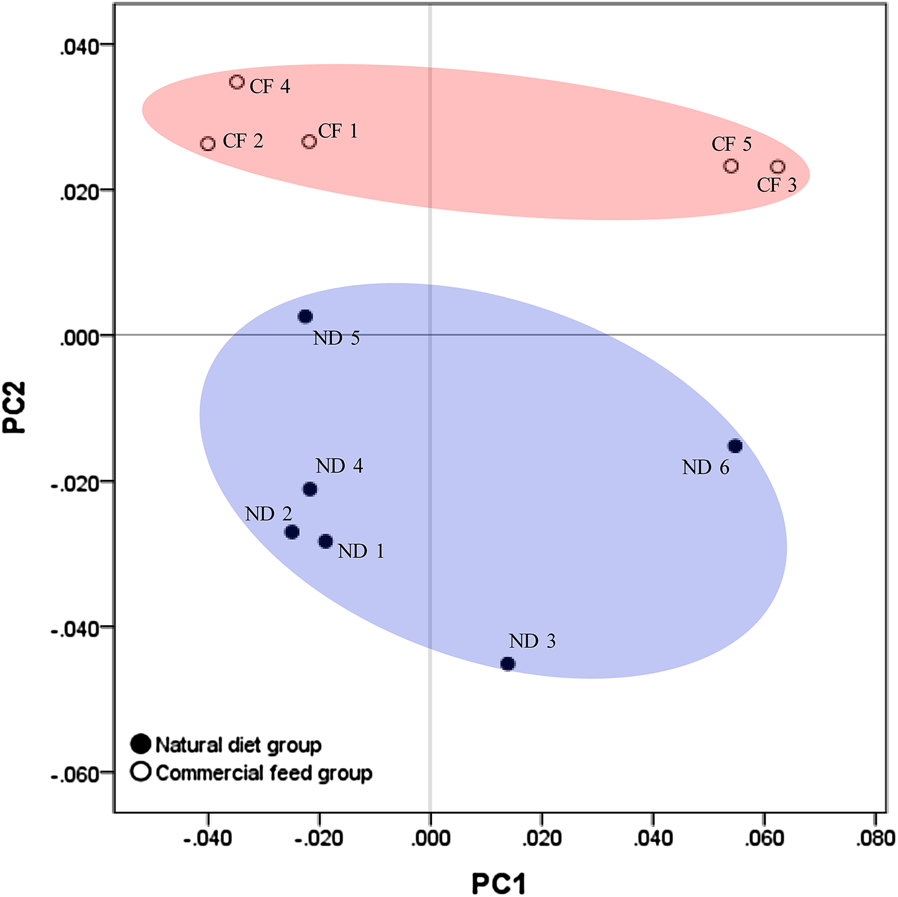
Principal component analysis (PCA) plot of the two diet groups. Beta diversity based on the Fast UniFrac distance matrix. The microbiota of the two diet groups showed pronounced differences in the PCA plot

PCA plots based on the Fast UniFrac distance metric were used to compare the composition of microbiota in the two animal groups. Upon PCA analysis, no difference in PC distribution was seen along PC 1, but a significant difference was observed along PC 2 (*p* = 0.004). The commercial feed group clustered together; the natural diet group also clustered together (Fig. 3). The raw data of PCO vectors for all samples are shown in Additional file 3.

### The core gut microbiota of dogs fed a natural diet or a commercial feed

At the phylum level, we identified eight different bacterial phyla in 11 dog samples; the core microbiota in the natural diet group comprised *Firmicutes*, *Bacteroidetes*, *Fusobacteria*, *Actinobacteria*, and *Proteobacteria*, and in the commercial feed group, these were *Firmicutes*, *Bacteroidetes*, *Proteobacteria*, and *Actinobacteria* (Table 2). These core gut microbiota constituted more than 99% of the total microbiota in each group; the predominant core microbiota at the phylum level was *Firmicutes*, followed by *Bacteroidetes*, regardless of the diet type.

**Table 2:** The core gut microbiota of dogs fed a natural diet or a commercial feed, at phylum, family, and species level

	Natural diet group	Mean	SEM	Commercial feed group	Mean	SEM
Phylum	Firmicutes	64.17	9.83	Firmicutes	73.33	14.19
	Bacteroidetes	19.89	9.79	Bacteroidetes	17.32	10.07
	Fusobacteria^a^	13.58	4.58	Proteobacteria	8.67	4.42
	Actinobacteria	1.50	0.67	Actinobacteria	0.65	0.59
	Proteobacteria	0.86	0.25			
Family	Lachnospiraceae	31.92	7.50	Lachnospiraceae	46.04	14.47
	Bacteroidaceae	17.59	9.81	Bacteroidaceae	16.49	9.58
	Clostridiaceae	16.12	8.77	Enterobacteriaceae	8.40	4.31
	Fusobacteriaceae^a^	13.55	4.57	Clostridiaceae	4.56	1.94
	Peptostreptococcaceae	7.62	3.11	Streptococcaceae	3.27	3.25
	Veillonellaceae	4.51	3.64	Allobaculum_f	2.84	0.92
	Streptococcaceae	2.43	1.72	Peptostreptococcaceae	2.56	1.55
	Prevotellaceae^a^	2.24	2.19	Enterococcaceae	2.32	1.40
	Coriobacteriaceae^a^	1.46	0.66	Coprobacillus_f	1.67	0.59
	Allobaculum_f	0.51	0.14	Veillonellaceae	1.02	1.01
	Enterococcaceae	0.37	0.23	Porphyromonadaceae^b^	0.79	0.79
	Enterobacteriaceae	0.35	0.12	Bifidobacteriaceae^b^	0.59	0.58
	Ruminococcaceae^a^	0.30	0.13	Sutterella_f^b^	0.24	0.23
	Coprobacillus_f	0.11	0.05			
Species	*Clostridium perfringens* ^a^	8.90	5.07	*Ruminococcus gnavus*	18.49	6.39
	*Clostridium rectum* ^a^	7.99	2.38	*Bacteroides vulgatus*	8.20	6.58
	EU465331_s^a^	5.76	2.19	*Escherichia coli* group	7.30	3.74
	ADLB_s^a^	5.59	2.11	GQ493555_s^b^	7.24	3.45
	*Ruminococcus gnavus*	4.60	1.25	GQ179695_s^b^	3.34	1.89
	*Clostridium hiranonis* ^a^	3.69	2.38	GL872355_s	2.52	1.39
	*Bacteroides*_uc	3.44	2.18	DQ795137_s^b^	2.49	1.34
	EF403475_s^a^	2.99	1.45	*Bacteroides*_uc	2.29	1.34
	GL872355_s	2.65	0.52	*Bacteroides dorei* ^b^	2.26	2.17
	*Bacteroides vulgatus*	1.98	1.03	*Clostridium difficile* ^b^	1.81	1.32
	*Clostridium*_uc	1.71	0.94	*Blautia*_uc	1.44	0.79
	*Fusobacterium*_uc^a^	1.66	0.64	*Ruminococcus*_g6_uc	1.36	0.54
	*Megamonas*_uc^a^	1.57	1.41	*Clostridium ramosum* ^b^	1.05	0.41
	*Streptococcus equinus* group^a^	1.47	1.43	*Lachnospiraceae*_uc_s	0.96	0.30
	*Clostridium sordellii* ^a^	1.44	0.95	*Escherichia*_uc^b^	0.76	0.41
	*Bacteroidaceae*_uc_s	1.33	0.79	*Bacteroidaceae*_uc_s	0.67	0.39
	*Blautia*_uc	1.28	0.41	JH590969_s^b^	0.56	0.38
	*Lachnospiraceae*_uc_s	1.22	0.14	*Clostridium paraputrificum* ^b^	0.51	0.14
	*Dorea*_uc	1.15	0.26	*Clostridium*_g6_uc^b^	0.41	0.13
	*Eubacterium tenue* ^a^	0.70	0.59	*Eubacterium*_g1_uc^b^	0.39	0.15
	*Fusobacterium varium* ^a^	0.65	0.24	*Dorea*_uc	0.39	0.13
	*Fusobacteriaceae*_uc_s^a^	0.57	0.21	*Clostridium*_uc	0.34	0.13
	*Ruminococcus*_g6_uc	0.53	0.13	*Enterobacteriaceae*_uc_s^b^	0.24	0.12
	*Clostridiaceae*_uc_s	0.54	0.27	*Anaerostipes caccae* ^b^	0.18	0.15
	EF401353_s^a^	0.51	0.05	*Hungatella*_uc	0.17	0.09
	*Clostridium*_g4_uc	0.50	0.21	*Romboutsia sedimentorum* ^b^	0.14	0.10
	FJ370676_s^a^	0.46	0.23	*Clostridium*_g4_uc	0.14	0.10
	*Veillonellaceae*_uc_s^a^	0.43	0.36	*Clostridiaceae*_uc_s	0.13	0.03
	*Eubacterium dolichum* ^a^	0.37	0.14	*Coprobacillus*_f_uc_s^b^	0.13	0.05
	EU772949_s^a^	0.29	0.13	*Allobaculum*_f_uc_s^b^	0.12	0.04
	*Escherichia coli* group	0.29	0.11			
	*Peptostreptococcaceae*_uc_s^a^	0.25	0.08			
	*Clostridium glycyrrhizinilyticum* ^a^	0.24	0.16			
	*Hungatella*_uc	0.18	0.06			
	FJ681620_s^a^	0.17	0.13			
	EF400787_s^a^	0.15	0.08			
	*Prevotella*_uc^a^	0.10	0.10			

Sequence data of taxon that did not match the existing standard strains were deposited in public database (http://www.ezbiocloud.net)

^a^The core microbiota of the natural diet group, but not the commercial feed group

^b^The core microbiota of the commercial feed group, but not the natural diet group

At the family level, 81 families were identified; among these, 14 families comprised the core microbiota in the natural diet group and constituted more than 99% of the total microbiota; in the commercial feed group, 13 families formed the core microbiota and constituted ca. 90% of the total microbiota (Table 2). Only 10 bacterial families from the core microbiota were shared by the two groups.

At the species level, 594 bacterial species were identified; among these, 37 species formed the core microbiota in the natural diet group and comprised ca. 67% of the total microbiota; in the commercial feed group, 30 species formed the core microbiota and comprised ca. 66% of the total microbiota (Table 2). Only 14 species from the core microbiota were shared by the two groups.

### Differences in the taxonomic composition of core gut microbiota of the two diet groups

**Fig. 4:**
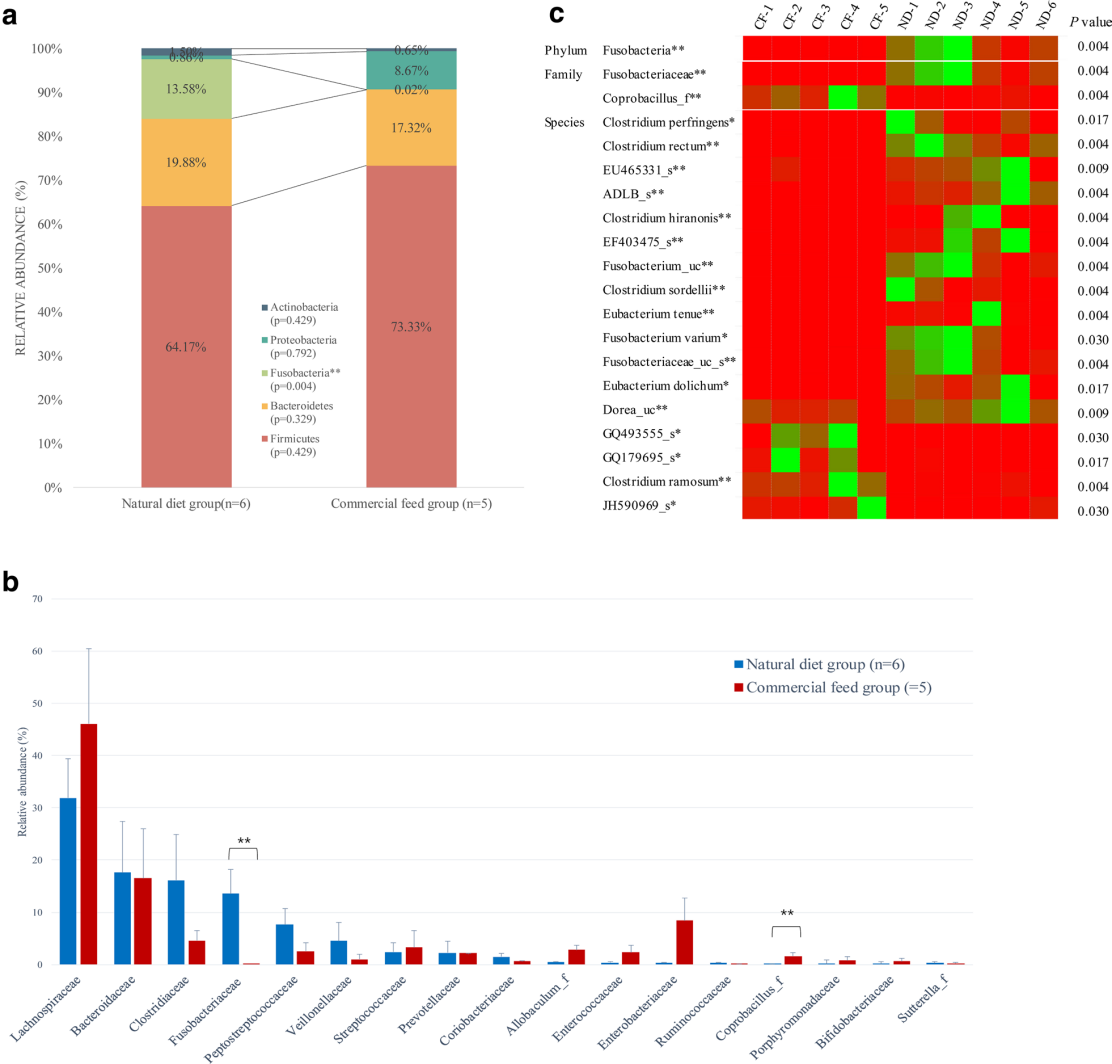
Differences in the taxonomic composition of core gut microbiota in the two diet groups. ND, natural diet group; CF, commercial feed group. *p < 0.05; **p < 0.01. The relative abundance of taxa differing between the two groups at the (**a**) phylum and (**b**) family levels is shown. **c** A heat map of the differences in the taxonomic composition of the core microbiota of the two groups. Red color, low abundance; green color, high abundance. Sequence data of taxon that did not match the existing standard strains were deposited in public database (http://www.ezbiocloud.net)

Differences in the taxonomic composition of core gut microbiota of the two diet groups were analyzed. The raw data of taxonomic composition at the phylum, family, and species level for all samples are shown in Additional file 4. At the phylum level, the abundances of Fusobacteria were different in the two groups; Fusobacteria were more abundant in the natural diet group than in the commercial feed group (*p* = 0.004; Fig. 4a). At the family level, *Fusobacteriaceae* was more abundant in the natural diet group than in the commercial feed group (*p* = 0.004), while *Coprobacillus*_f was more abundant in the commercial feed group than in the natural diet group (*p* = 0.004; Fig. 4b). At the species level, the abundance of 30 species was different in the two groups. In particular, *Clostridium ramosum* (*p* = 0.004) and *Anaerostipes caccae* (*p* = 0.009) were more abundant in the commercial feed group; *Clostridium perfringens* (*p* = 0.017), *Clostridium rectum* (*p* = 0.004), *Clostridium hiranonis* (*p* = 0.004), *Clostridium sordellii* (*p* = 0.004), *Eubacterium tenue* (*p* = 0.004), *Fusobacterium varium* (*p* = 0.030), *Eubacterium dolichum* (*p* = 0.017), and *Clostridium glycyrrhizinilyticum* (*p* = 0.017) were more abundant in the natural diet group (Fig. 4c). Differences in the taxonomic composition of the gut microbiota at the phylum, family, and species levels in the two groups are presented as a heat map in Fig. 4c.

## Discussion

To the best of our knowledge, the current study is the first to investigate the effect of long-term diet on the gut microbiota of dogs fed a natural diet compared with ones fed a commercial feed that have been actually applicated by dog owners by identifying the core microbiota up to the species level and comparing the differences in the gut microbiota between the two diet groups using NGS technology.

In the current study, the Illumina MiSeq platform and EzBioCloud database were used to analyze the gut microbiota of the dog. Among the various NGS systems, the Illumina MiSeq platform generates long and high-quality sequence reads, with the lowest error rates; it is also the most cost-effective platform, and hence suitable for small investigations [29–33]. The Illumina MiSeq platform was therefore chosen from the available NGS systems for the current study. In general, it is known that classification up to the species level may not be possible in MiSeq, because the species-level classification system of the reference database is not sufficient, rather than limitations of sequencing. In the case of SILVA and RDP, which are widely used, classification information is provided only up to the genus level, and the database is not updated periodically. However, the EzBioCloud database has a total of 78,870 taxa information (a total of 17,903 species with valid species names), which is more systematic, accurate, and periodically updated [34]. Therefore, data analysis using the EzBioCloud database allowed us to classify correctly up to the species level. The results of this study were similar to some but not all previous studies that employed the Illumina MiSeq platform to investigate the dog gut microbiota. In a study by Sandri et al. [19], the gut microbiota of dog was found to be composed of Firmicutes (43%), Bacteroidetes (19.8–26.9%), Fusobacteria (4.7–11%), and Proteobacteria (1.3–4.3%); in another study [35], it was found to be composed of Firmicutes (84.4% of all sequences), Bacteroidetes (2.9%), Fusobacteria (3.2%), Proteobacteria (7.8%), and Actinobacteria (1.7%). These differences might be due to individual variation of gut microbiota and differences in the animals. In particular, the gut microbiota is highly affected by the host genotype and environmental exposure, i.e., conditions that are difficult to standardize [36]. The inconsistency might also be attributable to the different DNA extraction kits employed. Previous studies have revealed that DNA yield, quality, and integrity, and the microbial community results vary depending on the DNA extraction kits used [37, 38]. The number of OTUs identified in the current study was nonetheless similar to that of other studies (range 129–242); furthermore, the Good’s library coverage in the current study was higher than that in other studies, which suggested that our results might reflect the actual bacterial gut community of dogs enrolled in this study.

In this study, diets were found to have a greater extent on the gut microbiota than other factors. No specific tendencies were observed when each sample was categorized by other factors (breed, gender, age, and weight of the dogs); however, there were pronounced differences in beta diversity, i.e., the measure of group comparison according to dietary types. Specifically, the samples mostly clustered together between the two diet groups based on PCA analysis (Fig. 3). In addition, significant differences were observed between the two diet groups in the number of OTUs, species richness, and evenness (Fig. 2). According to alpha diversity, i.e., species diversity, the richness estimates, and diversity indices of the microbiota of the natural diet group were significantly higher (*p* < 0.05) than those in the commercial feed group. Differences in the core microbiota at the phylum, family, and species levels were also observed between the two groups. The core microbiota comprised shared organisms found in the majority of individuals [39]. In the current study, core microbiota accounted for more than 99 percent at the phylum level, more than 90 percent at the family level, and more than 66 percent at the species level and could have a different impact on host health. Thus, the diet might indeed be responsible for the differences in alpha diversity, beta diversity, and the core microbiota. These differences might be due to differences in the way the two diet types were manufactured and differences in the main ingredients of the two diets. Generally, commercial feeds contain controlled nutrients and controlled microorganisms, because they undergo formal manufacturing processes, including compression through high temperature and high pressure and microbial monitoring. However, natural diets do not go through any manufacturing process and are fed to dogs as raw, so that more nutrients and microorganisms in the natural habitat are absorbed into the gut of the dog. In addition, these two diet types had differences in the main ingredients. The commercial feeds given to dogs recruited for the current study contained crude protein (18–21% of total content), crude fat (8–10%), crude fiber (3–5%), and crude ash (7%), with 10% of moisture. Based on these values, the main ingredients of the commercial feed were carbohydrates (up to ca. 50%). On the other hand, the main ingredients of the natural diet, which consisted of bones and raw meat, were crude protein (30–52%) and fat (11–50%), regardless of the meat type [40]. A previous study of the human gut microbiota revealed that there was a difference in gut microbiota between individuals with protein/fat-based eating habits and individuals with carbohydrate-based eating habits, because of the differences in microorganisms required for the digestion of carbohydrates, proteins, and fats [7]. By analogy, in the current study, the microbiota of dog would have changed depending on the most frequently consumed ingredients, i.e., the carbohydrate-based commercial feed and the protein- and fat-based natural diet. Moreover, the differences in beta diversity and the core microbiota observed herein were consistent with the results of other studies that demonstrated that long- or short-term diets play a substantial role in shaping human gut microbiota [7, 8].

From the perspective of microbial infection, this study suggests that the potential risk of opportunistic infection could be higher in dogs fed a natural diet than in dogs fed a commercial feed. Previous studies have revealed that dogs fed a natural diet are more likely to be exposed to bacterial contamination (ca. 30–50% of *Salmonella* spp. and 50% of *E. coli* group) and could be at a greater risk of foodborne illness than dogs given a commercial feed, because there was no regulation for microbial monitoring in raw meat and dogs fed a natural diet was more likely to be exposed to contaminated raw meat [14, 17, 18]. Furthermore, the presence of these opportunistic microorganism could pose a threat to public health, through dissemination of infectious agents to the owner or other animals whose immune system is weakened [15]. In the current study, the proportion of *C. perfringens* and *F. varium*, which are opportunistic microorganisms, was higher in the natural diet group than in the commercial feed group (Fig. 4c). *Clostridium perfringens* is usually found in the gut of human and animals, as a member of their normal flora [41]. Nevertheless, *C. perfringens,* which may infect the host after ingestion of contaminated raw beef or chicken, could cause necrotic enteritis, diarrhea, and gas gangrene [42, 43]. Furthermore, in the past, *C. perfringens* was the third most common bacterial foodborne illness in England and Wales; *C. perfringens* is still recognized as a foodborne pathogen [44, 45]. *F. varium* is also found in the gut of dogs as a member of the normal gut flora; however, under certain circumstances, i.e., if the microbial composition of *F. varium* was altered, or if contaminated with soil or feces, it might cause colon cancer, intra-ocular infections, and conjunctivitis [46, 47]. Specifically, in a mouse inoculation test, *F. varium* was found to be associated with ulcerative colitis [48]. However, in the current study, *Salmonella enterica* and pathogenic *E. coli,* which were previously detected in culture-based studies, were not evident from the NGS data [14, 18]. This discrepancy is probably because of the detection limit of the NGS system [49]. Culture-based protocols enrich the targeted microorganisms and control the growth of other microorganisms; on the other hand, NGS-based methods reflect the current distribution of gut microbiota without the need for any microbiological processing.

This study provides the basis for gut microbiota studies based on dietary type in dogs. However, because this study was conducted with healthy dogs, we cannot elaborate on the direct relationship between the health status and differences in microbiota according to diet type; we only discuss the risk of opportunistic infection. Therefore, to elucidate the correlation between health and diet type, follow-up studies need to be conducted with diseased dogs.

## Conclusion

This study is the first to analyze the association between dog gut microbiota and long-term diet (i.e., natural diet and commercial feed) using the Illumina MiSeq platform. Pronounced differences were detected in the microbiota of the two diet groups. Differences in the core microbiota at the phylum, family, and species levels were observed between the two groups. The microbiota of the natural diet group was characterized by higher richness and diversity compared with the commercial feed group. Samples from each group mostly clustered together according to the diet type. In addition, dogs fed a natural diet could be at a higher potential risk of opportunistic infection than dogs fed a commercial feed. Collectively, these results indicate that diet likely affects the microbiota, thereby playing a key role in animal health. This study provides the basis for gut microbiota studies based on dietary type in dogs; furthermore, this study, together with follow-up studies, could be used to ultimately devise an appropriate diet for dogs.

## Additional files

**Additional file 1.** PCR amplification conditions and primer sequence for bacterial DNA amplification.

**Additional file 2.** The raw data of diversity indices for all samples.

**Additional file 3.** The raw data of PCO vectors for all samples.

**Additional file 4.** The raw data of taxonomic composition at the phylum, family, and species level for all samples.

## Abbreviations

GI: gastrointestinal
NGS: next-generation sequencing
PCR: polymerase chain reaction
CD-HIT: cluster database at high identity with tolerance
PCA: principal coordinate analysis
OTUs: operational taxonomic units
